# Maternal vaccination: shaping the neonatal response to pertussis

**DOI:** 10.3389/fimmu.2023.1210580

**Published:** 2023-07-12

**Authors:** Maiya Callender, Eric T. Harvill

**Affiliations:** Department of Infectious Diseases, College of Veterinary Medicine, University of Georgia, Athens, GA, United States

**Keywords:** pertussis, maternal vaccination, Tdap, DTaP, neonatal immunity, window of opportunity, vaccines

## Abstract

Antepartum maternal vaccination can protect highly sensitive newborns before they are old enough to receive their own vaccines. Two vaccines are currently recommended during pregnancy: the flu vaccine and the Tdap vaccine against tetanus, diphtheria, and pertussis. Although there is strong evidence that maternal vaccination works to protect the offspring, limitations in the understanding of vaccines and of maternal transfer of immunity compound to obscure our understanding of how they work. Here we focus on the example of pertussis to explore the possible mechanisms involved in the transfer of protection to offspring and how these may impact the newborn’s response to future exposure to pertussis. For example, Tdap vaccines induce pathogen specific antibodies, and those antibodies are known to be transferred from mother to the fetus *in utero* and to the newborn via milk. But antibodies alone have modest impact on pertussis disease, and even less effect on colonization/transmission. Maternal immune cells can also be transferred to offspring and may play a direct role in protection from disease and/or influence the developing neonatal immune system. However, some of the transferred immunity may also blunt the offspring’s response to subsequent vaccination. In this review we will summarize the protection conferred to offspring by maternal vaccination against pertussis and the likely mechanisms by which protection is transferred, identifying the many knowledge gaps that limit our most effective application of this approach.

## 1 Introduction

Maternal vaccination provides important immunity in terms of generating pathogen specific immune components for the newborn (1). Antepartum vaccinations against tetanus, via the tetanus (TT) or the tetanus and diphtheria (Td) vaccines have been recommended since the 1960s (2). Currently it is recommended to vaccinate during pregnancy with the Influenza vaccine (during flu season), and the Tdap vaccine. Antibodies transferred from mother to offspring during pregnancy *in utero* and afterwards through breast feeding appear to be sufficient to limit tetanus and diphtheria. But immunity to pertussis is considerably more complex, and although maternal vaccination appears to be quite effective, the mechanisms of transferred protection are unlikely to be simply antibody-mediated and are poorly understood (3–5).

*Bordetella pertussis* (*Bp*) is a gram-negative bacterium (6) that causes an estimated 24-48 million cases of vaccine-preventable disease worldwide each year (3, 4). Symptoms can range from asymptomatic upper respiratory infection in fully immunized adults to severe pulmonary disease, which may be accompanied by characteristic paroxysmal coughing, post-tussive vomiting, bronchopneumonia, pulmonary hypertension, hypoxia, and death (4). While people of all ages may develop severe pertussis disease, newborns are the most susceptible population. Of the estimated 170,000 annual deaths caused by pertussis, over 50% of those occur in children under the age of 1 year (6, 7).

Due to the global impact of pertussis, two main vaccine formulations have been developed to combat this disease. The first was the whole cell pertussis (wP) vaccine, which was approved and distributed starting in the 1940s. While it proved very effective in preventing disease, rare reports of severe side effects and high reactogenicity led to the development of vaccines with less severe side effects (8). The acellular pertussis (aP) vaccines, comprised of 1-5 detoxified antigens, were introduced in the 1980s, and by the mid-1990s, many industrialized countries converted to these safer formulations (8) In many countries, aP vaccines are combined with tetanus toxoid and diphtheria toxoid to make up “DTaP” and “Tdap” vaccines. DTaP is primarily given to children under the age of 6 as a part of a 5-shot administration regimen. This schedule starts at two months old, leaving newborns vulnerable to severe disease until immunity can be generated.

Booster Tdap vaccination of mothers and close family members, has been highly recommended as a “cocooning” strategy to maximize the protection of newborns prior to receiving the first DTaP at two months of age. The primary aim of this strategy is to mitigate the transmission of *Bordetella pertussis* (*Bp)* to susceptible newborns. Initially, mothers were vaccinated postpartum but recently, this strategy was updated to have mothers receive the vaccine antepartum to generate anti-*Bp* antibodies that can be transferred from mother to child *in utero* during the later stage of pregnancy (9). While there has been evidence of protection conferred by these maternal antibodies, we do not yet know all the ways that vaccinating mothers against *Bp* can impact the newborn’s immune development and later response to *Bp* exposure.

Even though the aP vaccine is safe to administer during pregnancy (9), it has only been shown to prevent disease but not colonization in adults (10). Within the past two decades, studies have shown that aP vaccines can effectively prevent pertussis but enable asymptomatic infection and transmission to vulnerable hosts (11, 12). aP vaccine-induced protection wanes relatively rapidly and is of the less effective mixed Th1/Th2 response (13, 14). aP-induced antibodies are known to transfer from mother to child, but these maternal antibodies alone do not appear to be sufficient for full protection and have poorly understood effects on preventing disease/carriage or transmission for newborns. Maternal antibodies have also been reported to reduce the newborn’s ability to generate their own vaccine induced antibodies (15). These observations are still relatively new, and the mechanisms are not fully understood.

## 2 Protection conferred by maternal vaccination

### 2.1 Cocooning strategy

**Figure 1:**
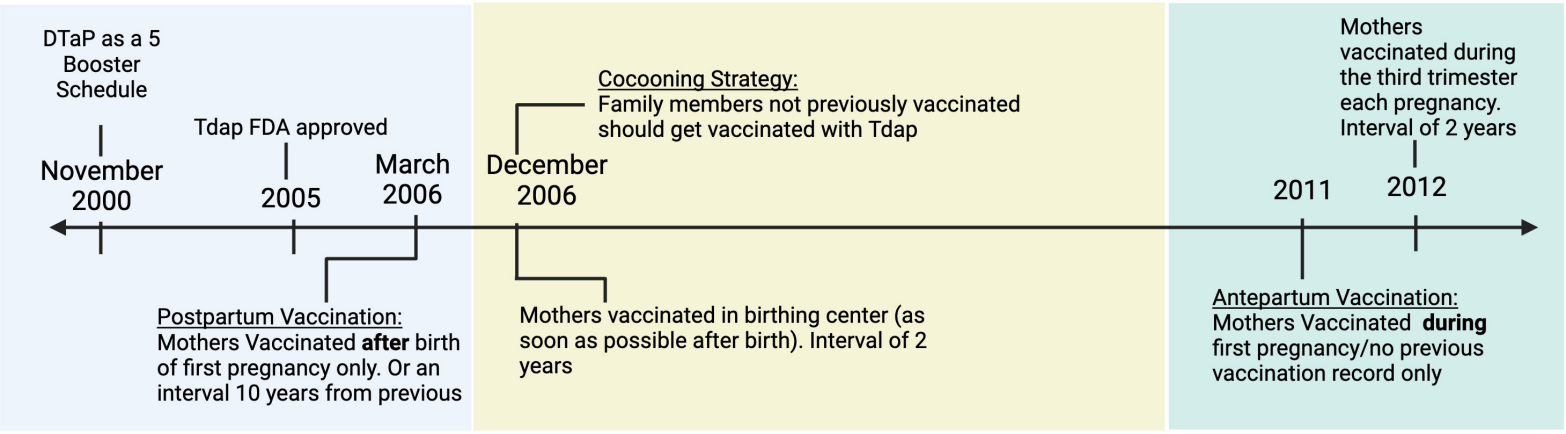
The timeline of recommendations from the Advisory Committee on Immunization Practices for administering the Tdap vaccine for Pregnant Women and Close Families. Created with Biorender.com.

Even though preterm vaccinations against Tetanus and Diphtheria (Td) had been recommended for many years, this form of vaccination against pertussis did not seem feasible until the Boostrix (Tdap) vaccine was approved in 2005. Boostrix was a less potent formulation of the DTaP vaccine meant to boost the *Bp*-specific-antibody titers observed to wane in older children and adults. Once the Tdap was approved in the United States, the Advisory Committee on Immunization Practices (ACIP) recommended it for mothers and close family members of newborns (16) (**Figure 1**). One of the first initiatives was a Cocooning strategy. This strategy had mothers receiving the Tdap vaccine postpartum before leaving the birthing center and close family members receiving the vaccine two weeks before meeting the baby, protecting vulnerable newborns by reducing transmission.

For the cocooning strategy to be effective, the aP vaccine would have to generate sterilizing herd immunity, preventing *Bp* colonization in close contacts of vulnerable infants. This is not possible with the current aP vaccine. aP vaccines reduce colonization in the lungs but don’t prevent colonization of the nasal cavity, allowing for shedding and silent transmission from mother or other caregivers to newborn (12, 17). Aside from the immunological ineffectiveness, the logistics of getting all close members of the family vaccinated proved difficult, limiting the success of this method of protection (16, 18).

### 2.2 Transfer of protection

Starting in 2011, in an attempt to boost immunity, a new strategy was developed as the primary method for newborn protection. Expecting mothers were advised to receive the Tdap vaccine during pregnancy (16) (**Figure 1**). This update allowed for anti-*Bp* antibodies generated by the mother to then be transferred to the child *in utero*. Antepartum maternal vaccination against *Bp* has been shown to induce high titers of pertussis-specific antibodies in newborns (19). These high titers are comparable to those in the vaccinated mothers showing the high efficiency of antibody transfer mechanisms. To achieve these high titers, mothers are vaccinated toward the beginning of the last trimester (27-36 gestation weeks) of pregnancy when the transfer of antibodies via the placenta is at its highest (20). *Bp*-specific IgG has been detected in the cord blood (21) and serum of newborns (22). *Bp*-specific IgA has also been detected in vaccinated mothers’ colostrum and newborns’ gut (23). These isotypes are the most associated with maternal vaccination, and their presence in the newborn highlights the effective transfer of pathogen specific antibodies from mother to child. Vaccinating mothers during pregnancy has been reported to decrease the chances of severe pertussis in babies by around 80%. A large retrospective study by Kaiser Permanente in California showed that maternal Tdap vaccination was 91% effective in protecting against pertussis during the first two months (24). Other clinical studies have inversely correlated pertussis-related hospitalization in infants with maternal vaccination status (25). While there has been an association between low hospital admission rates and higher maternal vaccination rates, such clinical studies cannot distinguish between protection conferred by herd immunity (preventing exposure), transfer of antibodies, cells or other signals. In fact, Tdap vaccines do not fully protect the mother from infection, and antibodies alone are known to have limited effect on *Bp* colonization, growth and transmission (26). Maternal antibodies have been shown to decrease the newborn’s immune response to subsequent vaccinations, also known as “blunting”, which could enhance susceptibility to outbreaks at a later time in childhood (27). Due to these potential downstream effects, it is important to better understand how current maternal vaccination strategies actually work to protect offspring.

In adult pertussis models, antibodies are hypothesized to perform a multitude of roles regarding clearance and protection. These include but are not limited to neutralizing bacterial toxins, inhibiting the binding of extracellular bacteria to respiratory epithelia, or antibody dependent uptake via macrophages and neutrophils occurring locally in the lungs (14). While high titer antibodies are associated with decreased chances of severe disease, there is no definitive correlation with sterile protection against infections by *Bp* (12).

Pertussis toxin (PT), a secreted protein from *Bp* has been associated with leukocytosis, severe pathology, immunosuppression and inflammation (28, 29) and is considered a key component of the acellular vaccine. Due to its importance, maternally derived anti-PT antibodies have been a focus of many maternal vaccination studies. The presence of high pertussis toxin IgG titers in the cord blood of newborns is used as justification for maternal vaccinations, as it is correlated with a reduction in newborn infections (30–32), although the mechanism of protection remains unclear.

Animal models have the potential to allow mechanistic studies of immunological components transferred from mothers to offspring, and the specific effects of each. Experimental models have included mice (33), non-human primates (34, 35) as well as neonatal pigs (33, 36). Using a baboon model, maternal antibody presence has been linked to reducing pathology associated with experimental challenges (35). While maternal vaccination of baboons with aP was sufficient to prevent disease in offspring, colonization was not prevented (35), distinguishing immunity from the disease from sterilizing immunity. Gaillard et al. (33) utilized a murine model to show that maternal vaccination protects offspring from *Bp* challenge in terms of reduced colonization in the lungs. The power of the immunological tools in the mouse model makes it feasible to more fully dissect the immunological mechanisms involved.

## 3 Maternal immune components and transfer

### 3.1 Maternal antibodies

**Figure 2:**
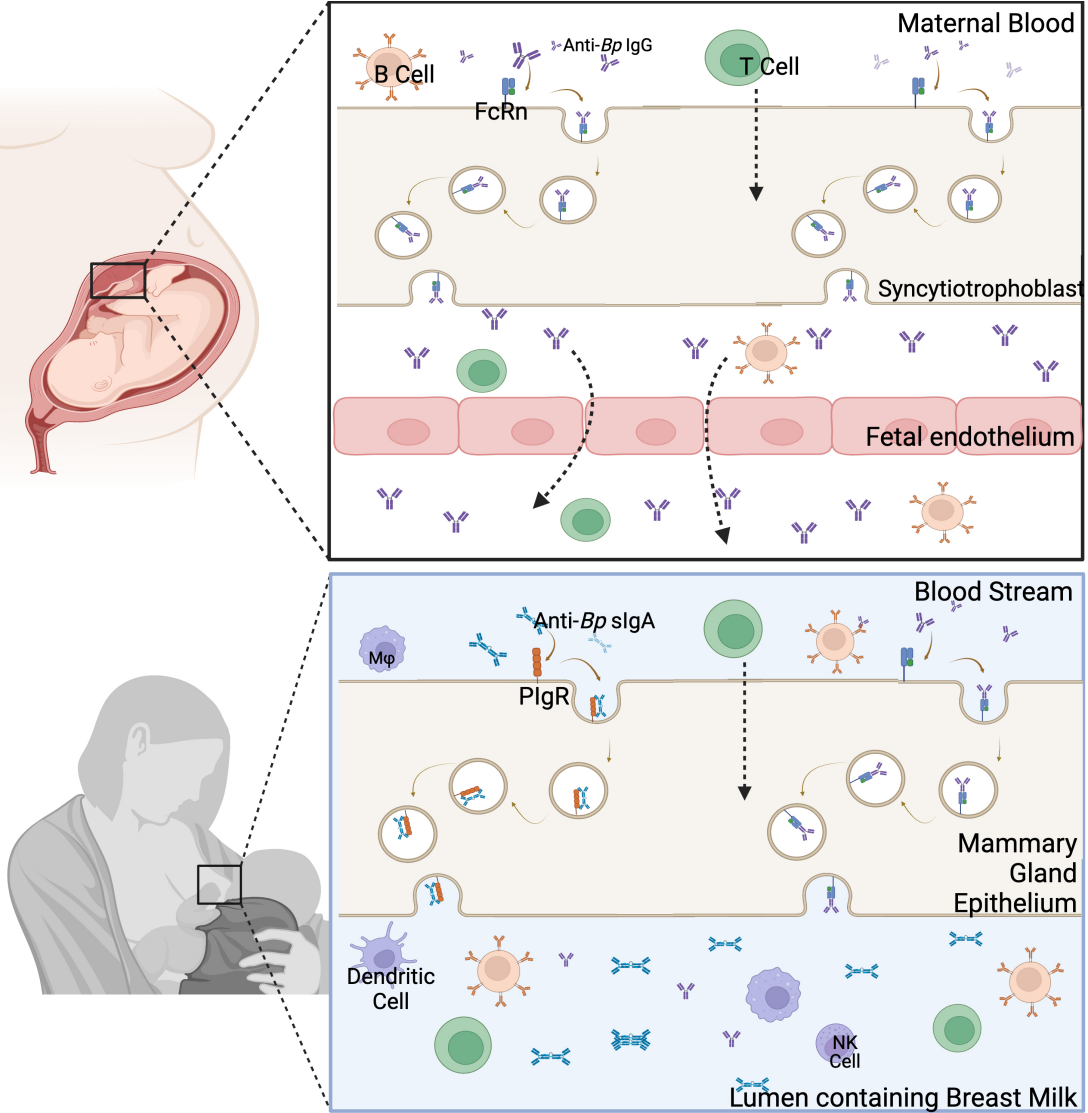
Main components of the two pathways of maternal immune component transfer. **(A)** Immune components are transferred from the blood of the mother through the placenta to the fetus. **(B)** The transfer of immune components through the breast milk occurs through the mammary gland epithelia to the lumen containing breast milk. Created with Biorender.com.

Maternally-derived immune components are vertically transferred antenatally via the placenta and perinatally via breast feeding. Exposing mothers to pertussis during pregnancy allows for pathogen specific antibodies to be transferred from mother to child (37). IgG is capable of crossing through the immune-privileged site of the uterus by binding with the neonatal Fc receptors (FcRn) on the placenta (38–40). At this interface, pathogen-specific IgG from the mother can be transferred to the fetus, providing added protection to the infant’s naïve immune system. This mechanism is pH-mediated, so once the pH is lowered, IgG binds to the FcRn and is then endocytosed by the syncytiotrophoblast to reach the fetal endothelium (**Figure 2A**) (41). The four subclasses of IgG are not equal in their binding and transfer across the placenta. IgG1 has the highest affinity for the FcRn (41–43). The following preferential order has been recently shown to be the IgG1 followed by IgG3, then IgG4 and IgG2 (42). These subclasses all have slightly different functions, so their relative presence in the newborns may give hints to the roles of maternal IgGs in protecting offspring from disease.

This transfer of antibodies does not end with birth. Pathogen-specific IgA and, to a lesser extent, IgG and IgM are passed to the newborn through the breast milk/colostrum allowing for the continued transfer of protective immune components during the first few months of life. Secretory IgA (sIgA) is transferred into the breast milk after binding to polymeric Ig receptors on the apical side of the breast tissue (**Figure 2B**) (39, 44), and sIgG is passed through the breastmilk using the FcRn, similarly as seen during placental transfer (44). Breast milk-derived antibodies may play an important role in mucosal immunity in newborns. sIgA and sIgG from breast milk have been shown to be present in the mucosal tissues of newborns (44). These antibodies from breast milk have been linked to protecting against gastrointestinal and respiratory illnesses (44–47). Anti-*Bp* antibodies have been found in the stool samples of preterm newborns fed either donor or mother’s milk up to 22 days postpartum. This study measured sIgA, sIgM, and sIgG titers of anti-PT and anti-Filamentous Hemagglutinin Antigen (FHA). It was found that anti-FHA IgG, anti-PT IgG, and IgA titers were consistent or increased in the gastrointestinal contents compared to the breast milk from the mother (23). A strong local mucosal response, as seen by high secretory IgA and serum IgG titers, has been shown to decrease *Bp* colonization in the upper respiratory tract (48–50). These results suggest that breast milk-derived antibodies can play an important role in mucosal immunity against pertussis. But even though antibodies are the main marker for maternal vaccination induced protection, using animal models, both B and T Cells are required for *Bp* clearance (14) and neither humoral nor cellular immunity alone is sufficient for full protection.

### 3.2 Maternal microchimeric cells

At the maternal-fetus interface, cells generated in the mother/fetus can be transferred from one to the other (51). These cells are known as microchimeric cells. There are many ways that microchimerism can occur, but pregnancy is the most common (51, 52). Maternal Microchimeric Cells (MMc), specifically, are cells from the mother that are present in the organs and tissues of infants and have been shown to persist throughout infancy and even into adulthood (53–55). MMc can be involved in innate and adaptive immune responses and may include, but are not limited to, B cells, T cells, and monocytes (38, 54, 56). The cells from the mother are transferred to the child via the uterus and through breast milk/colostrum, but the actual transfer mechanism is undefined (38) (**Figure 2**). Using models with immune deficient offspring, MMc have been shown to be able to fill the immune gaps in the neonatal immune system (52, 57). They have also been shown to improve immunity by providing protection against certain diseases (53, 58, 59). Maternal vaccinations have even been shown to increase MMc in infants (60). Even though the mechanistic role of MMc in protection has yet to be defined, due to the recent vast improvement in technology (53), we are able to track MMc in the neonatal tissues, define their role, and then apply what is learned to boost the neonatal immune system and protect against newborn diseases like pertussis.

Stelzler et al. (53) recently utilized an allogenic mating murine model to detect MMc. By mating mice with different CD45 and MHC Class I H-2D profiles, they were able to detect homozygous cells in the heterozygous offspring. With their model, they were able to show that MMc presence correlates with a decrease in Murine Cytomegalovirus (MCMV) in the bone marrow, lungs, and salivary glands. They also utilize PCR to detect deletions and insertions polymorphisms (DIPS) in human offspring specific to their mother. With this, they could correlate MMc presence in the cord blood with a decrease in childhood respiratory illnesses (53). This model and other systems for detecting microchimeric cells will be useful for exploring how vaccines induce pathogen specific MMc and their impact on the neonatal response to pathogens like *Bp*.

## 4 Influence of maternal vaccination on neonatal immune responses

Children under six months are highly susceptible to severe *Bp* infections. The pathology of those infections includes leukocytosis, bacterial pneumonia, and pulmonary hypertension. Maternal intervention during pregnancy can impact this response, skewing the immune system toward a different immune response that may be more protective. Maternal vaccination has been shown to impact neonatal immune development *in utero*. B and T cell immune responses to Influenza have been detected from cord blood of infants of vaccinated mothers. Newborn’s response to antigen transferred *in utero* was seen by the presence of anti-Fluzone and anti-matrix specific IgM as well as hemagglutinin (HA) specific CD4+ T Cells in the cord blood (61). This shows that there is a unique window of opportunity to teach the neonatal immune system to combat severe infections better.

### 4.1 Impacts on neonatal immunity

Maternal exposure to pathogens and microbes has been shown to impact the neonate’s response regarding altering the cellular profile of newborns (62). This can be beneficial as it may be a way to educate the newborn’s immune system about pathogens before exposure. Newborns were once regarded as having an underdeveloped immune system, with their adult immune system developing over time. The emerging view is that newborns have a unique immune system specific to their neonatal challenges (63–66). The neonatal immune environment skews anti-inflammatory to prevent unnecessary inflammation as the newborn is introduced to commensal bacteria. While this can be beneficial in protecting the newborn from unnecessary inflammatory damage, the anti-inflammatory response is not as effective against certain diseases. Pregnant mothers also have a Th2-skewed anti-inflammatory response (67), which is essential so that the body does not attack the developing fetus. This natural skew is vital to understand as the two *Bp* vaccine platforms available (wP and aP) prime different T cell responses. The wP vaccine primes a very protective Th1 response, which has been shown to be the most effective in the clearance of *Bp* and inducing immunity, while the aP primes a not-as-effective mixed Th1/Th2 profile (14).

The natural skew toward anti-inflammatory response is critical to note as maternal vaccination with the Tdap vaccine has interestingly been shown to induce a more protective pro-inflammatory profile in newborns. This is seen by reduced levels of the cytokines IL-10 and IL-4 and increased levels of the cytokines IL-2 and IL-12 (68). There has also been evidence that the change in the monocyte profile can boost protection against disease (68). Maternal vaccinations may also modulate the T cell profile of neonates. Effector cytokines associated with a Th2 response were decreased after the DTaP boost, showing that maternal antibodies may play a role skewing the newborn’s immune response toward a mixed Th1/Th2/Th17 response versus a Th2 response (69). Since antibody titers, specifically IgG, are the current gold standard for measuring vaccination efficacy, more research into newborn cellular response induced by maternal vaccination is needed, as it may link to the protective nature of this method of vaccination.

### 4.2 Immunoblunting induced by maternal antibodies

**Figure 3:**
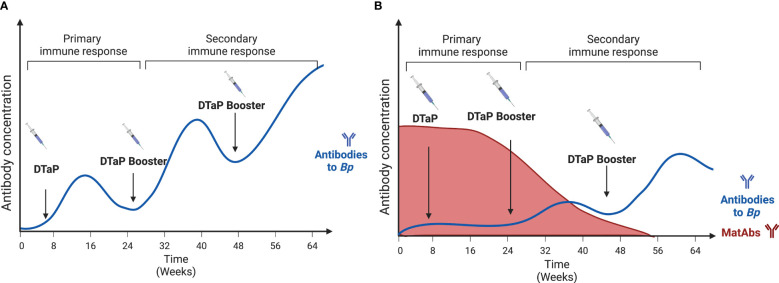
The blunting effect of maternal antibodies on the development of DTaP antibody responses by the offspring following vaccination. **(A)** represents the normal antibody response to vaccines. Each booster induces higher concentrations of anti-*Bp* antibodies. **(B)** Represents the offspring’s antibody response in the presence of Maternal antibodies. Here the response is delayed until the Maternal antibodies’ levels have depleted. Created with Biorender.com.

While maternal vaccination has been shown to provide valuable protection for newborns, it has also been reported to blunt the newborn response to vaccinations (15). Infants whose mothers were vaccinated with the Tdap vaccine have been shown to have a limited DTaP vaccine response in terms of lower antibody titers. In a study following infants’ vaccinations over the first year of their lives, infants whose mothers received the Tdap vaccine had a reduced antibody titer for diphtheria and pertussis (15). This effect continues until the maternal antibody levels wane (**Figure 3**). Maternal vaccination against *Bp* has been shown to reduce anti-PT and anti-FHA titers in newborns after their first couple of vaccinations (27, 70, 71). Some studies have shown that this effect disappears after boosters that occur post age 2, likely due to the decay of maternal antibodies by that age (72). This blunting effect does seem to be inconsistent amongst the different formulations of the vaccine (27, 73, 74).

Clinical studies focusing on how maternal vaccination can interfere with infant vaccine responses have shown conflicting results. These studies measure the antibody titers to the different components of the aP vaccine: Pertussis toxin (PT), Pertactin (PRN), Filamentous hemagglutinin antigen (FHA), and Fimbriae 2/3 (FIM2/3). Englund et al. (75) originally showed that maternal antibodies lead to a modest impact on pertussis antibodies in infants who received the wP vaccine. This trend, where wP-vaccinated infants are the most impacted by maternal exposure, was also shown recently in a study comparing the antibody responses from over 150 infants (70). However, a study in Pakistan showed no blunting effects on antibody titers to PT, PRN, FHA, or FIM2/3 (76). This inconsistency and variability show the limitations of clinical studies with different number of individuals studied and the diverse vaccine formulations.

Animal studies using murine and porcine models have shown that a pertussis toxoid vaccine does blunt the antibody response in offspring (77), although the mechanism is still debated. The Antibody Feedback inhibition theory involves the inhibition of antibody response through the overabundance of antigen-specific antibodies (72). So, for *Bp-*specific blunting, high concentrations of *Bp-*specific maternal antibodies would lead to the infant’s reduced anti-*Bp* antibody production. Other hypotheses include the inhibition of B cell responses. One of those is epitope masking, as maternal antibodies may cover the epitopes on vaccine antigens, preventing B cell recognition (72). Another B cell inhibition theory states that inhibition occurs through the crosslinking of B cell receptors and Fc*γ* receptor III via maternal antibodies (72). A recent study has provided an alternative explanation that maternal antibodies alter plasma cell and memory B cell differentiation. These B Cells in the germinal center exhibit different V_H_ and V_K_ genes and have altered isotype switching ability, further showing how maternal antibody presence has effects on the newborn’s developing responses (78).

To the relevancy of this blunting, there have been suggestions to alter the vaccination schedule to optimize the newborn’s response to vaccines. The suggestions range from increasing the number of boosters to delaying the vaccine schedule (30, 77). Doing this can offset any potential lasting effects of a blunted vaccination response. Another suggestion is to take advantage of the protective effect of maternal vaccinations. By targeting mothers as an avenue for vaccinating newborns and developing better therapeutics, we can boost the immune response and possibly even better teach the newborn’s immune system to face pathogens. Since maternal vaccination with the Tdap vaccine limits the newborn’s response to the first few administrations of the DTaP vaccine, it is essential that we truly understand the protection conferred by maternal vaccination.

## 5 Future directions

Future directions in this field should aim to explore maternal vaccine options that confer protection from disease and colonization for the mother. By doing both, we can allow for herd immunity to protect the most susceptible. By focusing on vaccinating mothers during pregnancy, it will be possible to protect mothers and susceptible newborns with just one shot. These new *Bp* vaccines need to induce protection and cannot impact neonatal response to other vaccines. Some techniques to bypass the blunting effects of antepartum maternal vaccination include the utilization of different adjuvants and altering the vaccination route by using mucosal vaccines on the newborn (77, 79). Using different adjuvants, Polewicz et al. (80) showed, using both murine and porcine models, that offspring developed a superior antibody protection and a more balanced Th1/Th2 response compared to traditional formulations.

Many factors that could potentially affect how the advantages of maternal vaccination are conferred across populations have yet to be addressed. There is substantial variability in human populations that is poorly represented in uniformly housed, inbred mouse populations. These factors can range from genetics and health to socioeconomic and ethnic backgrounds. Some populations in the United States have lower vaccinations rates due to a lack of access to care and medical information as well as there being vaccine hesitancy rooted in historic events. Lower maternal Tdap vaccination rates are seen among Hispanic and non-Hispanic Black populations therefore impacting seroprotection of the newborn, thus making these groups more vulnerable to infection (81–83). It is important to understand how these factors might combine to deprive some populations of the advantages of maternal vaccination is worth consideration and requires more study.

## 6 Conclusion

Maternal vaccination leads to the passive transfer of protective immune components to the newborn. These components have been linked to decreased pathology in *Bp* infections and can play a role in instructing and shaping the neonatal immune response to better protect against diseases. To fully understand these important effects, more research is necessary, especially concerning the efficacy of maternal vaccination in conferring protection to offspring. The recommendation for mothers to get vaccinated during pregnancy is less than two decades old. Clinical experience and experimental models have shown aP vaccination confers protection from disease but not colonization, possibly contributing to the increased asymptomatic transmission and vaccine evasion currently seen with circulating strains (11). Along with this, we must know how this method of vaccination impacts the developing immune system. The first few months of a newborn’s life is a prime window of opportunity to prepare the developing immune system to better fight against pathogens. Although we know that vaccines are safe for the pregnancy and development of the fetus, the long-term effects of this strategy are not fully understood.

## Author contributions

MC and EH conceived the review. MC and EH wrote the manuscript. MC generated the figures. Both authors contributed to the article and approved the submitted version.
